# Organoaxial gastric volvulus: a rare cause of an acute abdomen

**DOI:** 10.3205/iprs000130

**Published:** 2019-03-25

**Authors:** Katrin Bauer, Christof Keller

**Affiliations:** 1 Department for General, Visceral, Vascular, Thoracic and Pediatric Surgery, Kempten Clinic, Kempten, Germany

**Keywords:** gastric volvulus, upper-GI-obstruction, epigastric pain, vomiting

## Abstract

We report the case of a 65-year-old male patient with massive epigastric pain of sudden onset and vomiting due to an organoaxial volvulus of the stomach. We comment on the surgical management in our case and discuss etiology and therapeutic options of this rare entity.

## Zusammenfassung

Wir berichten über einen 65-jährigen Mann mit hochakutem Abdomen und starkem Erbrechen, ausgelöst durch einen organoaxialen Magenvolvulus. Wir kommentieren unser chirurgisches Vorgehen und diskutieren den gegenwärtigen Kenntnisstand bezüglich dieses seltenen Krankheitsbildes.

## Introduction

Gastric volvulus is a rare clinical entity and often difficult to diagnose. It can cause a sudden onset acute abdomen due to strangulation and gastric ischemia as well as chronic, unspecific symptoms like intermittent epigastric pain, recurrent vomiting and weight loss due to insufficient food intake. The severity of the symptoms depends on the kind of twisting and the rapidity of onset of the anatomic dissolution of the stomach [1], [2]. The formal definition of gastric volvulus comprises abnormal rotation of the stomach of more than 180 degrees with reversal of normal gastric anatomy and at least intermittent obstruction. The most frequently used classification system describes 3 types of gastric volvulus in decreasing order of frequency: organoaxial volvulus, mesenteroaxial volvulus and combinations of the aforementioned [1], [2], [3]. In organoaxial volvulus the stomach rotates along a cardiopyloric long axis and is often associated with concurrent hiatal hernia or trauma. In mesentericoaxial volvulus, the rotation occurs along an oblique short axis through the stomach, it is also associated with paraesophageal, hiatal hernia and can complicate an upside-down stomach [2]. Males and females seem to be equally affected by the condition. Gastric volvulus is more common in the elderly population and is in most cases organoaxial. In children, a mesentericoaxial rotation secondary to congenital diaphragmatic defects is seen more often. Therapy depends on the kind of rotation, rapidity of onset, clinical signs of ischemia and severity of obstruction and includes conservative, endoscopic and surgical treatment [1], [2], [3].

## Case description

A 65-year-old male patient (BMI 24 kg/m^2^) presented to our emergency department with severe epigastric pain of sudden onset and recurrent eruptive vomiting. He had never had any similar symptoms in the past. His medical history included allergic asthma, depression, appendectomy, urolithiasis and mild aortic stenosis. Abdominal examination revealed epigastric peritonism with a distended abdomen, bowel sounds were absent. Abdominal ultrasound was not informative due to massive amounts of air in the upper abdomen. Blood tests showed mild leucocytosis (10,4 Gpt/l) and a CRP-value of 13,75 mg/l. The other values including liver enzymes, INR, electrolytes, urea and creatinine were regular. 

**Figure 1:**
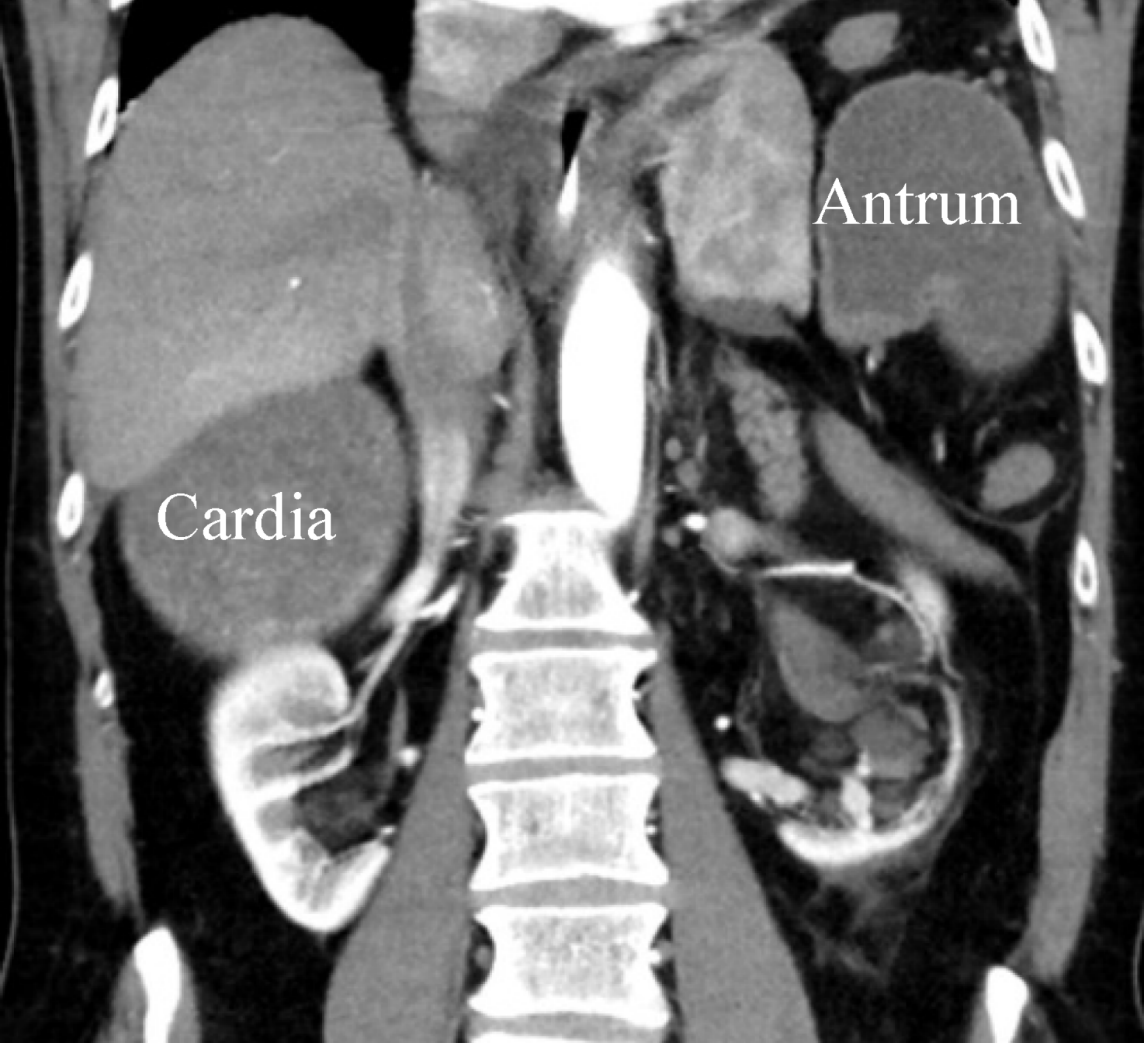
CT scan: dislocation of the cardia to the right lower epigastrium and the antrum to the upper left abdomen

**Figure 2:**
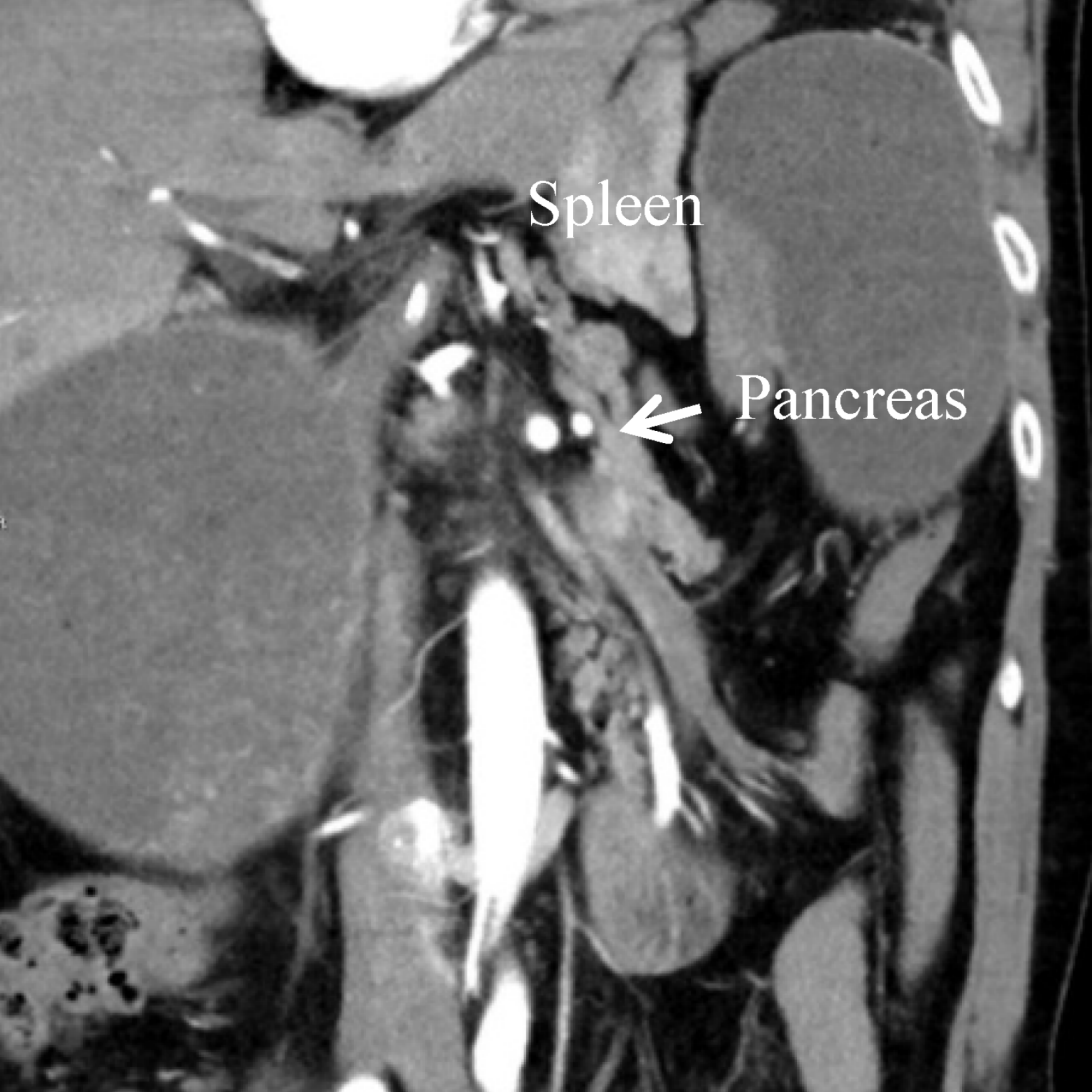
CT scan: spleen and pancreas tail shifted medially

**Figure 3:**
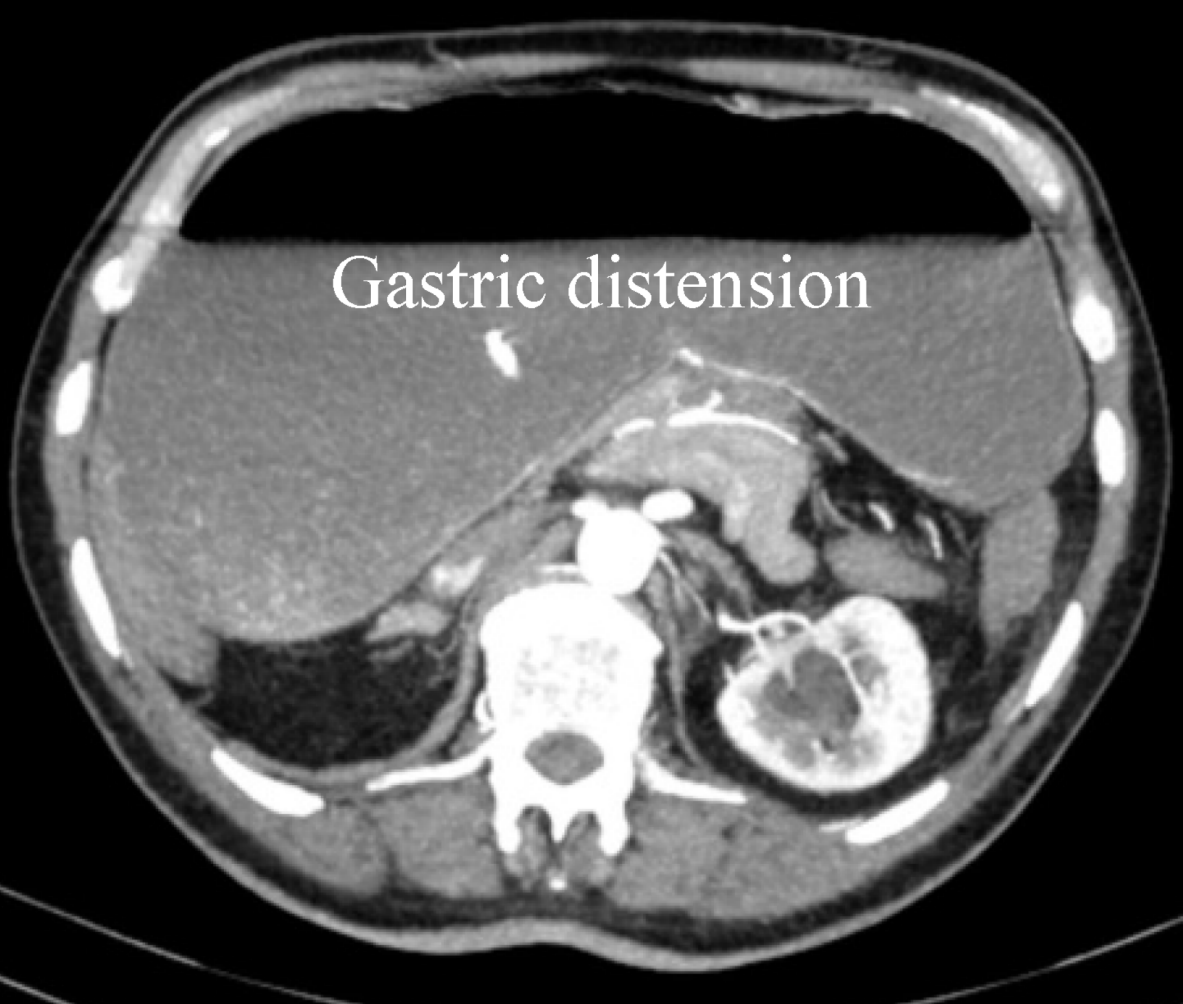
CT scan: massively distended stomach

An emergency CT scan of the abdomen revealed a dislocation of the anatomical cardia to the right lower epigastrium and the gastric antrum to the left upper side (Figure 1 (Fig. 1)), spleen and tail of the pancreas were shifted medially (Figure 2 (Fig. 2)). The stomach appeared massively distended (Figure 3 (Fig. 3)). There was no free gas in the abdominal cavity and there were no direct signs of gastric gangrene.

Because of the massive gastric distension and the very distressed condition of the patient we decided against an endoscopic intervention and performed an immediate exploration via laparotomy to avoid ongoing gastric gangrene. Intraoperatively the diagnosis of organoaxial volvulus was confirmed, there was ischemic congestion of the stomach without gangrene. After manual reposition the stomach appeared vital, a large bore gastric tube was inserted transorally and large amounts of air and non-digested food were decompressed. After that, we performed gastropexy of the gastric fundus to the left diaphragm with non-absorbable sutures. There was no major hiatal hernia or diaphragmatic defect. The patient made a full recovery and could be demitted 8 days after the emergency procedure.

## Discussion

Berti was the first to describe a gastric volvulus in a female autopsy patient in 1866 [4]. Depending on the etiology, gastric volvulus can be classified as primary (idiopathic) or secondary. Primary gastric volvulus occurs due to abnormalities of the gastric ligaments with abnormal or failed gastric fixation. Secondary gastric volvulus arises in the presence of local anatomic abnormalities including paraesophageal hernia, diaphragmatic hernia, phrenic nerve paralysis and atypical anatomy of adjacent organs and rupture. Other predisposing factors include diaphragmatic injury or surgery, congenital hernias and diaphragmatic eventration associated with phrenic nerve paralysis, left lung resection or pleural or intraabdominal adhesions. Both organoaxial and mesentericoaxial can lead to gastric gangrene due to local ischemia [1], [2]. The range of symptoms varies from incidental radiographic findings to life-threatening emergencies, depending on the rate of progression and extent of gastric rotation [3].

The Borchardt triad describes a clinical triad of acute gastric volvulus including severe epigastric pain with distension, vomiting and difficulty or inability to pass a nasogastric tube into the stomach [2].

The mortality rate of acute gastric volvulus is reported to be up to 50%, secondary to gastric ischaemia, perforation, and necrosis. Subtotal or total gastrectomy is proposed when the stomach appears gangrenous [1], [2].

In cases of early diagnosis and vital gastric structure, a reposition and gastropexy seems to be the appropriate treatment. Concurrent pathology of the diaphragm should be repaired, normally via a dorsal hiatoplasty [1], [2]. In high risk patients, endoscopic decompression and reduction may be an option. Successful endoscopic derotation manoeuvres have been described in the literature, even in acute gastric volvulus [5].

## Conclusion

Gastric volvulus is a rare entity that can present as a rare cause of intermittent epigastric pain with unspecific symptoms but also as a life-threatening acute abdomen, as presented in our case. While chronic gastric volvulus can also be treated conservatively, acute gastric volvulus is an abdominal emergency with high mortality rates and should be treated immediately by surgical intervention to avoid ischemic gastric necrosis.

## Notes

### Competing interests

The authors declare that they have no competing interests.
